# Pathological effect of arterial ischaemia and venous congestion on rat testes

**DOI:** 10.1038/s41598-017-05880-2

**Published:** 2017-07-14

**Authors:** Shuichi Hirai, Naoyuki Hatayama, Munekazu Naito, Kenta Nagahori, Shinichi Kawata, Shogo Hayashi, Ning Qu, Hayato Terayama, Sunao Shoji, Masahiro Itoh

**Affiliations:** 1 Department of Anatomy, Aichi Medical University, 1-1 Yazakokarimata, Nagakute, Aichi 480-1195 Japan; 2 Department of Anatomy, Tokyo Medical University, 6-1-1, Shinjuku, Shinjuku-ku, Tokyo 160-8402 Japan; 3 Department of Anatomy, Division of Basic Medical Science, Tokai University School of Medicine, 143 Shimokasuya, Isehara, Kanagawa Japan; 4 Department of Urology, Tokai University Hachioji Hospital, 1838 Ishikawamachi, Hachiōji, Tokyo 192-0032 Japan

## Abstract

Many studies on various organs have concluded that venous congestion (VC) causes severe organ dysfunction with elevation of oxidative stress relative to that of arterial ischaemia (AI). However, a comparison of the pathological effects of AI and VC on the testes has not been conducted. In this study, models of AI and VC and their reperfusion in rat testes, respectively, were developed and analysed. Testicular arteries or veins were interrupted for 6 h, re-perfused and kept for 4 weeks; the effects on the testes were then evaluated. Severe spermatogenic disturbances were observed at 4 weeks after reperfusion in AI but not in VC. At 6 h after blood flow interruption, oxidative stress was significantly increased and germ cells were severely damaged in AI compared with those in VC. RT-PCR analyses revealed that haem oxygenase-1, which exhibits anti-oxidative effects, and vascular endothelial growth factor-A, which exhibits vasculogenic effects, were significantly increased in VC but not in AI. Surprisingly, the results of our experiment in rat testes differed from those of experiments in previous studies performed in other organs. Oxidative stress in testes was more easily elevated by AI than it was by VC, explainable by the different experimental conditions.

## Introduction

In general, blood flow interruption (BFI) is either caused by arterial ischaemia (AI) due to arterial inflow interruption or venous congestion (VC) due to venous perfusion interruption. To investigate the effects of AI and VC, a number of experimental models have been used to examine various anatomical sites, such as the digestive tract, skin and spinal cord, and the results have shown that AI and VC exhibit different pathologies^1–5^. Macroscopically, it is known that digestive tracts with VC turn more dusky red than those with AI and that oxidative stress causes severe disturbance^4, 5^. In the skin and spinal cord, it has also been demonstrated that VC leads to more severe damage and harmful effects than AI^1–3^.

The testes require abundant blood flow for active spermatogenesis and testosterone secretion^6, 7^. Therefore, BFI easily leads to spermatogenic disturbance^8^. Testicular torsion is a particularly well-known condition that is accompanied by acute BFI. Many researchers have investigated the pathology of testicular torsion. It is known that acute BFI causes oxidative stress within the testes, which leads to spermatogenic disturbance^9, 10^. Moreover, it has also been found that apoptosis caused by the Fas/Fas-L and Bax pathways are related to germ cell death because of acute BFI^11–13^. A previous study investigated the testicular pathologies suffered from AI and VC until 2 h^14^. Further, BFI time is important for inducing the degree of testicular injury, and 6 h is a critical time for deciding surgical treatments for testicular torsion. However, investigation of testicular blood flows by clearly distinguishing AI from VC pathologies at 6 h has not yet been reported.

This study aimed at determining the respective pathological effects after reperfusion following AI or VC on the testes using an experimental animal model.

## Results

**Figure 1:**
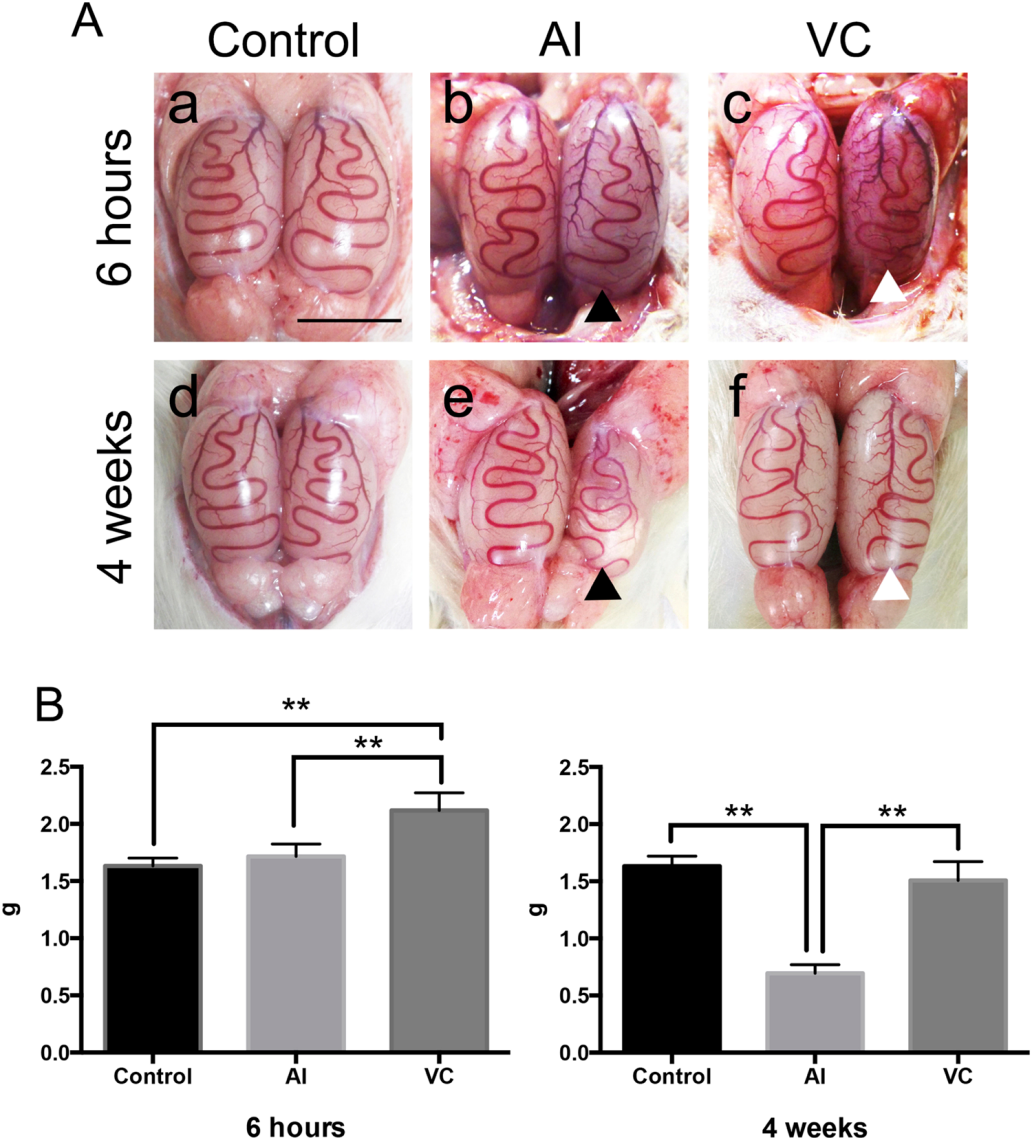
Macroscopic findings and organ weights of rat testes with arterial ischaemia (AI) and venous congestion (VC) and after their reperfusion. A: Macroscopic findings. (**a**) Control. (**b**) 6 h after AI. (**c**) 6 h after VC. (**d**) control after 4 weeks. (**e**) 4 weeks after reperfusion of AI. (**f**) 4 weeks after reperfusion of VC. The black arrowheads indicate the testes that had received AI and its reperfusion. The white arrowheads indicate the testes that had received VC and its reperfusion. Bar = 1 cm. B: Changes in testes weights in rats with AI or VC. Data are expressed as means ± SD, and the two-way analyses of variance and Tukey–Kramer tests were used for statistical analyses. *P* > 0.05 was considered as indicating a nonsignificant difference. **P < 0.01.

**Figure 2:**
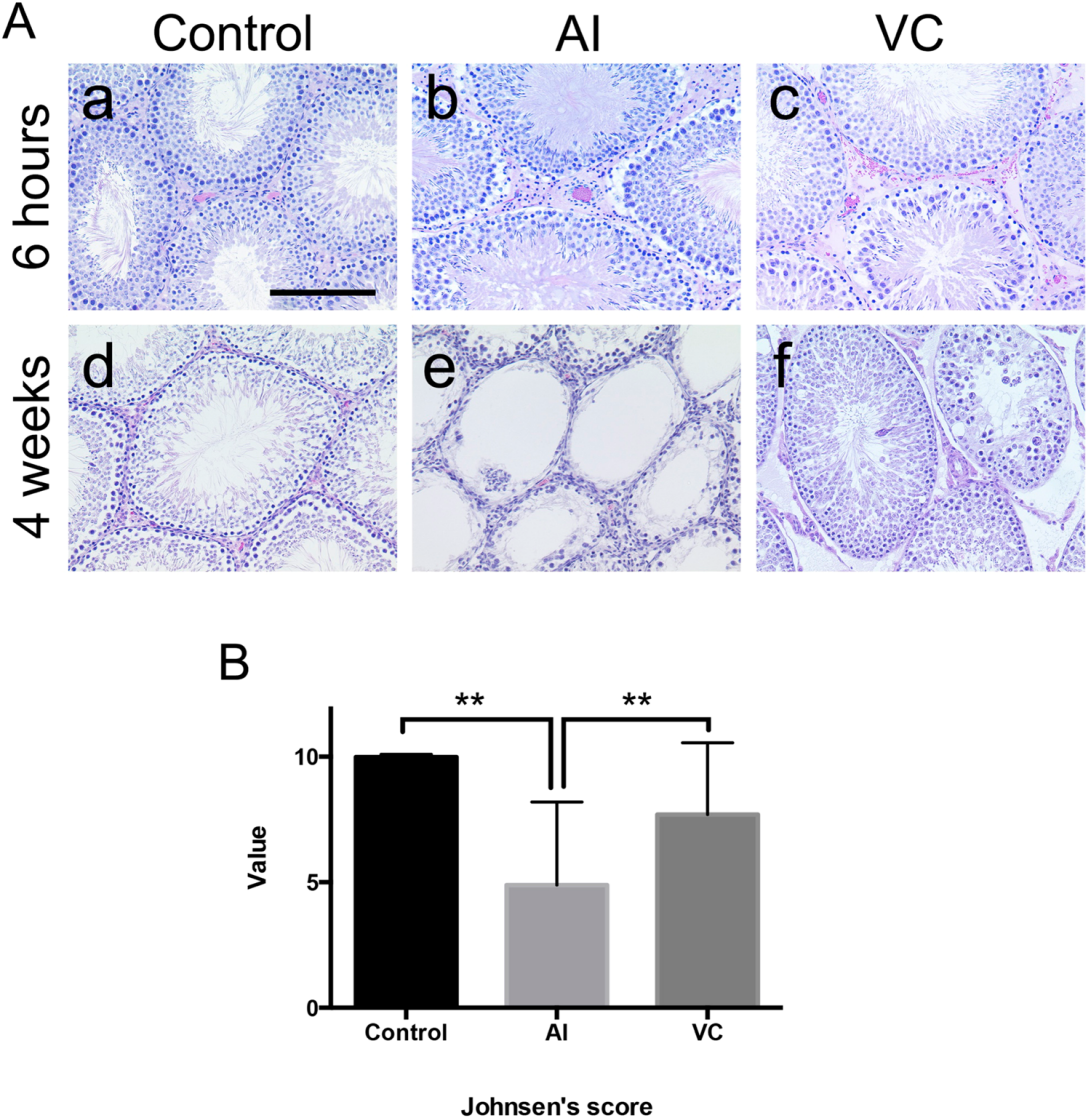
Histopathological changes in rat testes with arterial ischaemia (AI) and venous congestion (VC) and after their reperfusion. A: (**a**) Control. (**b**) 6 h after AI. (**c**) 6 h after VC. (**d**) control after 4 weeks. (**e**) 4 weeks after reperfusion of AI. (**f**) 4 weeks after reperfusion of VC. Interstitium area in (**c)** showing the haemorrhage. Bar = 50 μm. B: Degrees of spermatogenesis at 4 weeks after reperfusion of AI or VC. Data are expressed as means ± standard deviation, and the two-way analyses of variance and Tukey–Kramer tests were used for statistical analyses. *P* > 0.05 was considered as indicating nonsignificant differences. **P < 0.01.

The testes color in the AI group became significantly whiter at 6 h after BFI (Fig. 1Ab), while the testes color in the VC group significantly changed dusky red (Fig. 1Ac). Petechial haemorrhage appeared in the testes of the VC group but not in those of the AI group (Fig. 1Ab and c). A comparison of testis weight revealed that the testes of the VC group had become swollen and heavier compared with those of the AI group (Fig. 1B). Histologically, the stagnation of blood cells in the small vessels of the testes and expansion of the interstitium were noted in the testes of the VC group but not of the AI group (Fig. 2Ab and c). Moreover, a small amount of haemorrhaging in the interstitial tissue was found in the testes of the VC group but not of the AI group (Fig. 2Ab and c).

After 4 weeks of reperfusion, the left testes of the AI group were atrophied (Fig. 1Ae and B), whereas the left testes of the VC group did not change relative to that of the control group (Fig. 1Af and B). Histologically, in the AI group that had received reperfusion, severe spermatogenic disturbance showing maturation arrest or the Sertoli cell-only feature was observed (Fig. 2Ae and B). In contrast, only mild spermatogenic alterations were found in the VC group (Fig. 2Af and B).

**Figure 3:**
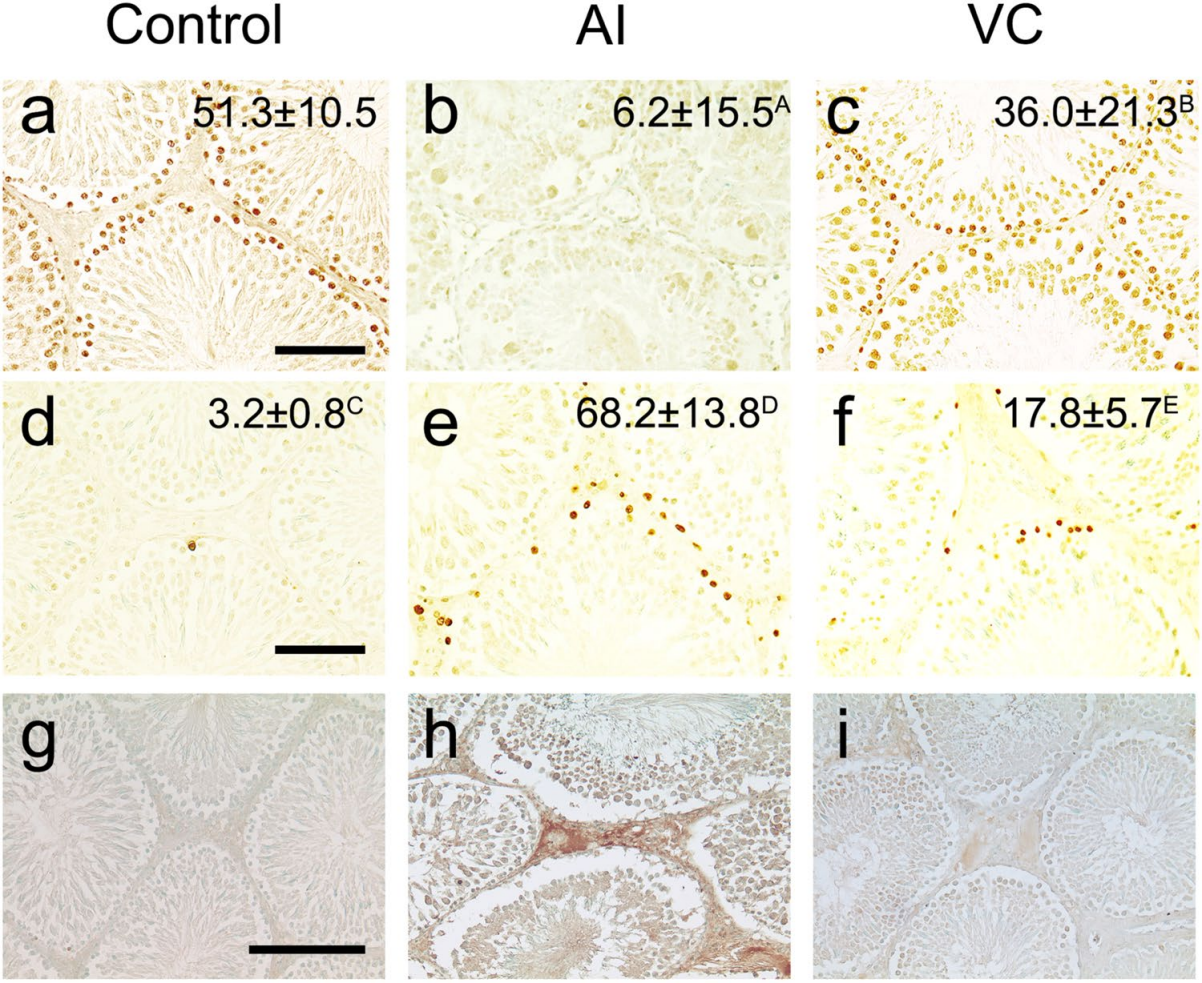
Proliferating cell nuclear antigen-positive cells (**a**–**c**), Tdt-mediated dUTP nick end labelling-positive cells (**d**–**f**) and 8-hydroxydeoxyguanosine (**g**–**i**) in the sections of rat testes with arterial ischaemia (AI) and venous congestion (VC) at 6 h. (**a**,**d** and **g)** Control group. (**b**,**e** and **h**) AI group. (**c**,**f** and **i**) VC group. Bar = 50 μm.

In the testes of the VC group at 6 h after BFI, many proliferating cell nuclear antigen (PCNA)-positive germ cells close to the basement membrane of the seminiferous tubules were detected, as in the control group (Fig. 3a and c). However, in the AI group, few PCNA-positive germ cells were observed (Fig. 3b). The number of these positive cells was significantly lower in the AI group (6.2 ± 15.5) than in the VC group (36.0 ± 21.3).

TdT-mediated dUTP nick end-labelling (TUNEL)-positive germ cells close to the basement membrane of the seminiferous tubules were occasionally detected in the testes of the control group (Fig. 3d). At 6 h after BFI, TUNEL-positive cells were more prominently detected within the seminiferous tubules and interstitial tissue in the testes of the AI group (Fig. 3e) than of the VC group (Fig. 3f). A significant increase in the number of TUNEL-positive cells in the seminiferous tubules was observed in the testes of the AI group (68.2 ± 13.8) relative to that in the VC group (17.8 ± 5.7) at 6 h after BFI (Fig. 3e and f). 8-OHdG could be evidently detected in the interstitium area of sections from the AI group but faintly detected in those from the VC group (Fig. 3h and i).

**Figure 4:**
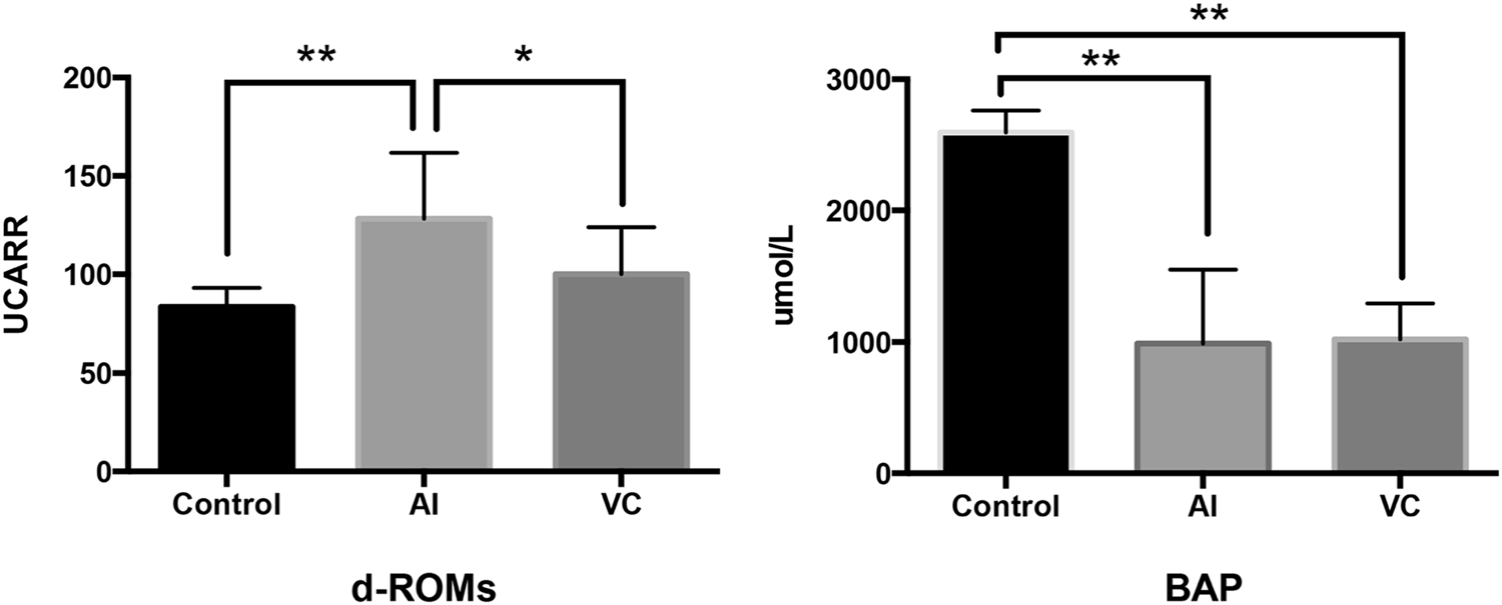
Reactive oxygen metabolite-derived compound (ROM) test and the biological antioxidant potential (BAP) test in rat testes with arterial ischaemia (AI) and venous congestion (VC) at 6 h. Data are expressed as means ± SD, and the two-way analyses of variance and Tukey–Kramer tests were used for statistical analyses. *P* > 0.05 was considered as indicating nonsignificant differences. *P < 0.05. **P < 0.01.

Total oxidative stress level (d-ROMs) was significantly higher in the AI group than in the control and VC groups (Fig. 4). Total antioxidant levels (BAP) were significantly lower in the AI and VC groups than in the control group (Fig. 4).

**Figure 5:**
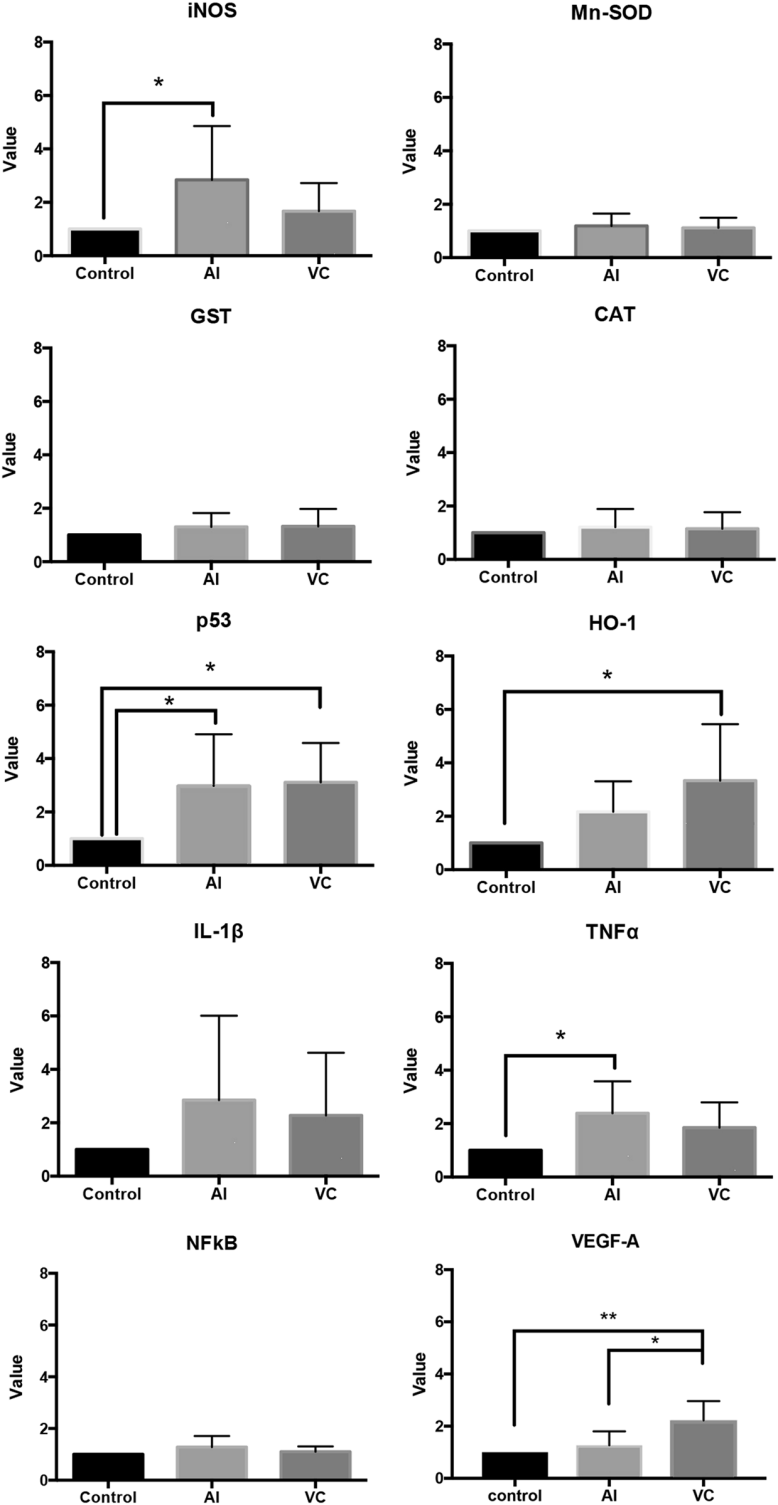
Expression of mRNAs of inducible nitric oxide synthase (iNOS), superoxide dismutase (SOD), glutathione-*S*-transferase (GST), catalase (CAT), p53, haem oxygenase (HO)-1, interleukin (IL)-1β, tumor necrosis factor (TNF)-α, nuclear factor kappa-light-chain-enhancer of activated B cells (NF-κB) and vascular endothelial growth factor (VEGF)-A from rat testes with arterial ischaemia (AI) and venous congestion (VC) at 6 h. Relative intensity was calculated after normalising the expression to that of the control (taken to be 1). Each bar represents the mean ± SD (*n* = 6). *P < 0.05. ***P < 0.005.

Real-time reverse transcriptase-PCR analyses revealed a large increase in the mRNA expression of inducible nitric oxide synthase (iNOS) and TNF-α in the AI group but not in the VC group (Fig. 5). The expression of superoxide dismutase (SOD), glutathione-*S*-transferase (GST), catalase (CAT), IL-1β and NF-κB mRNA did not show such changes in the AI and VC groups. In contrast, the expression of p53 increased in the AI and VC groups relative to the control group (Fig. 5). The expression of TNF-α significantly increased in the AI but not VC group. However, the protein levels of p53 were not significantly different among the control, AI and VC groups (Supplemental Fig. 2). Both mRNA expression and protein levels of haem oxygenase-1 (HO-1) prominently increased in the VC group but not in the AI group (Fig. 5, Supplemental Fig. 2). Further, VEGF-A expression significantly increased in the VC group compared with the control and AI groups (Fig. 5).

## Discussion

In this study, we developed a model of AI or VC and their reperfusion. At 4 weeks after reperfusion, severe spermatogenic disturbances were observed in the AI group, whereas only mild spermatogenic alterations were detected in the VC group. The results showed that the proliferative germ cells had decreased severely in the AI group relative to that in the VC group and that the dead germ cells had increased more in the AI group than in the VC group at 6 h after BFI. Moreover, the oxidative stress was more severe in the AI group than in the VC group.

A number of studies have compared the effects of AI and VC on various organs^1–5^. The results of these studies have concluded that VC causes more severe organ dysfunction than does AI^1–5^. Furthermore, reports involving experiments on the digestive tract and spinal cord have shown that VC increases intra-organ pressure much greater than does AI, which leads to oxidative stress and consequent cell damage^9, 15^. Huang *et al*. reported no pathological difference in the testicular injuries between the AI and VC groups until 2 h.(40) Indeed, the results of our experiment on rat testes demonstrated that there were little differences in testicular pathologies 2 h after interruption (Supplemental Table 1). However, surprisingly, AI group induced testicular severe damage compared with VC at 6 h. Moreover, the expression of TNF-α capable of inducing apoptosis and inflammation increased in the AI group but not in the VC group. In general, it is known that the testis is an easily congested organ because of its anatomical characteristics. The testicular blood vessels originate in the abdomen and course down through the inguinal canal as part of the spermatic cord on their way to the testes. Upward flow of blood in the veins is ensured by small one-way valves that prevent backflow. Defective valves or compression of the vein by a nearby structure can cause dilation of the testicular veins near the testes, which leads to the formation of a varicocele. The prevalence rate of varicocele is approximately 15% in healthy men and 40% in infertile men^16^. In patients with varicoceles, the collateral circulation chronically develops through the cremasteric vein and vas deference vein in scrotal organs and the spermatic cord^17^. In fact, surgical ligation or embolisation of the testicular vein is used in male infertility treatment for varicoceles^18, 19^. In contrast, we assumed that the collateral circulation did not work in our experimental model of acute BFI because the testes with VC obviously changed to dusky red, were swollen and exhibited petechial haemorrhage.

Our data demonstrated that oxidative stress in the testes was more easily produced by AI than by VC. Potentially toxic reactive oxygen species (ROS) are produced during normal cellular metabolism and clearly can be toxic to cells; nevertheless, it has been demonstrated that proper production of ROS was required for spermatogonial stem cell self-renewal^20, 21^. Therefore, the competition between ROS and antioxidant enzymes, such as SOD, GST and CAT, is essential in the testes^22^. Moreover, the ‘guardians’ of the genome, p53 and HO-1, could be useful to combat the effects of oxidative stress^23, 24^. In the present study, the total antioxidant capacity decreased in the AI and VC groups, and the intra-testicular mRNA expression levels of SOD, GST and CAT did not change in either group. Therefore, there was no difference in the antioxidant responses between the AI and VC groups. In contrast, the mRNA expression and protein levels of p53 prominently increased in the AI and VC groups, and that of HO-1 in the VC group significantly increased relative to that in the AI group. Our results raise the possibility that the testes have a mechanism that reduces the damage caused by the ROS accompanying VC. Previous studies have demonstrated that antioxidant and anti-inflammatory treatment modalities, such as colchicine and thymoquinone, help to reduce ischaemia-reperfusion injury in the detorsed testis^25, 26^. These modalities might be especially effective for ameliorating tissue damages in the pathological condition of AI. Moreover, this study demonstrated that the mRNA expression of VEGF-A significantly increased in the VC group compared with the control and AI groups (Fig. 5). A previous study reported the protective effect of VEGF on histological damage in testicular torsion^27^. VEGF-A expressions may important for preserving the spermatogenic activity in VC group.

In cases of testicular torsion, prolonged BFI has been shown to cause necrosis of the affected testis, which resulted in irreversible spermatogenic disturbance^8^. The degree of testicular injury has been correlated with predicted BFI time (i.e., whether it is ≤6 h), the extent of torsion observed intraoperatively and macroscopic findings in the affected testis. Orchiectomy is comprehensively decided using these findings. Thus, the decision on whether to perform orchiectomy depends heavily on the urologist’s experience^28^. In fact, in some testicular torsion cases in which ≥6 h has passed since symptom onset, surgery was not performed because of the potential for recovering testicular function^28^. Therefore, an accurate understanding of testicular cord torsion pathology is a clinically important issue. It has been reported that testicular torsion is mainly caused by AI^29^. However, because the arterial walls are thicker and more elastic than venous walls and arterial pressure is much greater than is venous pressure, VC occurs more easily than does AI. In reality, both AI and VC could exist in testicular torsion pathology. Our color histogram analysis revealed that petechial haemorrhage that appeared in the testes was cause by VC but not by AI and that there was no microscopic difference between the AI and VC groups immediately after 5 min of reperfusion (Supplemental Fig. 1). Our experimental findings suggest that in cases of testicular BFI, macroscopic findings are unsuitable indexes for predicting testicular function. Doppler evaluation has been shown to be useful for evaluating testicular blood flows in cases of testicular torsion^30–32^. For evaluation of prognosis of testicular function, our results support the adoption of onset time of testicular torsion or the measurements of testicular blood flows rather than the macroscopic findings.

## Conclusions

In a rat model of AI or VC and their reperfusion in testes, AI caused severe dysfunction with elevation of oxidative stress relative to that in VC. Subsequently, severe spermatogenic disturbances were observed in the AI group, whereas only mild spermatogenic alterations were detected in the VC group at 4 weeks after reperfusion. The total antioxidant capacity in both AI and VC is equivalent, but HO-1 in VC significantly increased relative to that in AI. The testes may have a mechanism that reduces the damage caused by ROS accompanying VC. Moreover, VEGF-A was also significantly higher in the VC group compared with the AI group and may have produced different spermatogenic pathology between AI and VC. This is the first report to compare the pathological effects of AI and VC on the testes and presents a new opinion, in addition to established theories, regarding acute BFI in testes.

## Methods

### Animals

Sprague–Dawley rats (8-week-old males; *n* = 42) were purchased from SLC (Shizuoka, Japan) and maintained at 22 °C–24 °C and 50–60% relative humidity with a 12-h light–dark cycle in the Laboratory Animal Center of Tokyo Medical University for 2 weeks before use. The handling and care of the rats conformed to the National Institutes of Health (NIH) guidelines for animal research, and all experimental protocols involving animals were approved by the National Research Institute for Child Health and Development Animal Care and Use Committee (Permit Number: S24018). All experiments involving animals were performed according to these guidelines and experimental protocols. All efforts were made to minimise animal suffering.

### Experimental designs

Ten-week-old male rats were deeply anesthetised with pentobarbital (65 mg/kg body weight) and kept under anesthesia by inhalation of isoflurane (ISOFLU^®^; Dainippon Sumitomo Pharma Co., Ltd.) using ventilation (air, 400–500 ml/min, isoflurane 1.5–2.0%; Univentor 400 anesthesia Unit; Univentor Ltd., Zejtun, Malta). Under anesthesia, the abdomen was opened and the blood flow of the testicular artery or vein was interrupted using Silver Neuro Clips (Muromachi Inc., Tokyo Japan). To examine the changes in testis color, the right testicular artery and left testicular vein were clipped. The testes subjected to AI or VC (*n* = 6 each) were observed at 2, 4 and 6 h. The testes were also observed at 5 min and 1 h after AI or VC for 6 h. Testicular artery or vein interruption caused a change in testicular color, so it was easy to determine the success of interruption (Supplemental Fig. 1A). We analysed the means of the histogram from the grey-scale image. Ischaemia was defined by a change in brightness of ≥10%, and congestion was defined by a change in brightness of <10% at 2 h after interruption. We analysed only the testicular samples that matched the definition (Supplemental Fig. 1B).

To evaluate the pathological effect of AI and VC on rat testes, the rats were divided into three groups as follows. The first group comprised rats that underwent a sham operation in which the abdomen was opened and closed (control group: *n* = 6). The second group comprised rats that underwent clipping of their left testicular artery at 6 h (AC group: *n* = 6). The third group comprised rats that underwent clipping of their left testicular vein at 6 h (VC group: *n* = 6). To examine reperfusion injury, rats that received reperfusion after AI (AC group: *n* = 6) or VC (VC group: *n* = 6) and control rats (control group: *n* = 6) were kept for 4 weeks. The rats were sacrificed under deep anesthesia at 6 h or 4 weeks after BFI. The testis weights were recorded, and histopathological samples were obtained from each group at 6 h and 4 weeks after BFI. The total RNA samples and interstitial fluid were collected from a part of the left testis from each group at 6 h.

### Light microscopy

The testes from each group at 6 h and 4 weeks after BFI (control group: *n* = 12; AI group: *n* = 12; VC group: *n* = 12) were fixed in 10% buffered formaldehyde for 5 days, washed, dehydrated in an ethanol series and embedded in paraffin. Sections 5-μm thick were then stained with Gill’s haematoxylin and 2% eosin Y. The histopathological changes were evaluated with the following Johnsen’s scoring system, ranging from a score of 1 (no germ cells in the seminiferous tubules) to 10 (complete spermatogenesis)^33^. The sections were analysed at 200× magnification by light microscopy. Twenty areas of 1 mm^2^ were randomly examined, and >200 round- or oval-shaped seminiferous tubules were counted in the testes of each group.

### Proliferating cell nuclear antigen staining assay

For detecting cell proliferative activity, samples from each group at 6 h after BFI (control group: *n* = 6; AI group: *n* = 6; and VC group: *n* = 6) were examined by immunohistochemical labelling for PCNA staining as described previously^34^. Sections were finally counterstained with methyl green. Negative controls were prepared by omitting the primary antibody from the staining procedures. For statistical analysis, 100 round or oval seminiferous tubules/animal were counted, and the number of seminiferous tubules containing PCNA-positive germ cells/100 seminiferous tubules [mean ± standard deviation (SD)] and the average number of PCNA-positive cells in a seminiferous tubule (mean ± SD) in each testis were determined.

### TUNEL-labelling assay

To detect apoptotic cells, samples from each group at 6 h after BFI (control group: *n* = 6; AI group: *n* = 6; and VC group: *n* = 6) were examined using a commercially available TUNEL staining kit (Apop Tag Plus Peroxidase *In Situ* Apoptosis Detection Kit; Serologicals, Purchase, NY, USA), as described previously^12^. For statistical analysis, 100 round or oval seminiferous tubules/animal were counted, and the number of seminiferous tubules containing TUNEL-positive germ cells/100 seminiferous tubules (mean ± SD) in each testis was determined. The average number of TUNEL-positive cells in a seminiferous tubule in each testis was determined.

### Immunohistochemical detection of 8-hydroxydeoxyguanosine

To detect oxidative stress, samples from each group at 6 h after BFI (control group: *n* = 6; AI group: *n* = 6; and VC group: *n* = 6) were examined using 8-hydroxydeoxyguanosine (8-OHdG) as the primary antibody. Deparaffinised and dehydrated sections were treated in a microwave oven at low power for 10 min in 0.01 M sodium citrate buffer (pH 6.0) to retrieve antigen, and endogenous peroxidase was inactivated using 3% hydrogen peroxide in methanol for 10 min and then blocked with blocking solution for 1 h at room temperature. Sections were then incubated, respectively, with a 1–20 dilution of mouse monoclonal antibody to 8-OHdG (Japan Institute for the Control of Aging, Fukuroi, Shizuoka, Japan) overnight at 4 °C. The incubation of biotinylated anti-mouse secondary antibody and horseradish peroxidase (HRP)-labelled streptavidin and staining with diaminobenzidine was performed according to the manufacturer’s instructions (Vector Laboratories, Burlingame, CA).

### Measurement of reactive oxygen metabolite-derived compound (d-ROM) and biological antioxidant potential (BAP) tests

In recent years, many methods have been developed for detection of both oxidant and antioxidant statuses. With good-to-excellent analytical performances, both d-ROM and BAP tests have been proven to be reliable and suitable methods for assessing oxidative stress, as indicated by numerous studies on humans and several animal species, including mammals^35–37^. Therefore, d-ROM and BAP tests were chosen in this study because of their accuracy in measuring the total oxidant stress and total antioxidant capacity, respectively, of the interstitial fluid. The interstitial fluid d-ROM and BAP levels in each group (control: *n* = 5; AI group: *n* = 5; and VC group: *n* = 5) were measured by the spectrophotometric method^38^ using the Free Radical Elective Evaluator (Diacron, Grosseto, Italy). The interstitial fluid was centrifuged, and 20- and 10-μl samples were used to measure d-ROM and BAP levels, respectively. d-ROM values were expressed in the arbitrary unit U. CARR^38^.

### Gene expression analysis

The testes were obtained from each group at 6 h after BFI (control group: *n* = 6; AI group: *n* = 6; and VC group: *n* = 6). Total RNA was isolated from the entire testis using a TRIzol RNA extraction kit (Invitrogen, CA, USA) according to the manufacturer’s instructions. cDNA was prepared according to a standard protocol (high-capacity cDNA archive kit; PE Applied Biosystems, Foster City, CA, USA), and the mixtures were stored at 80 °C until analysis. Real-time PCR of 3-ng cDNA was performed using a validated SYBR Green gene expression assay along with the SYBR Premix Ex Taq II (TaKaRa Bio Inc., Ohtsu, Japan) for measuring rat iNOS, SOD, GST, CAT, p53, HO-1 and glyceraldehyde-3-phosphate dehydrogenase (GAPDH). All primers used in this study are listed in Table 1. Quantitative real-time PCR was performed in duplicate in a thermal cycler dice real-time system TP800 (TaKaRa), and the data were analysed using the same system. The comparative Ct method (2DDCt) was used to quantify gene expression levels. Data on real-time PCR products were standardised to GAPDH, which was used as the negative control. To confirm the specific amplification of the target genes, each gene product was further separated on a 1.5% agarose gel to detect any single bands at the theoretical product sizes, and the dissociation curves were analysed to detect any single peak.

**Table 1:** Name and accession number of the target genes, listed together with the sequences of the specific primer pairs.

Gene	Accession number	Forward (5′–3′)	Reverse (5′–3′)
iNOS	NM_012611	GCAGGTTGAGGATTACTTCTTCCA	GCCCTTTTTTGCTCCATAGGAAA
SOD	NM_013671	GGCTCCCGGCACAACACAGCC	CCTCGTGGTACTTCTCCTCGGTG
GST	NM_013541	GTGCCCGGCCCAAGAT	TTGATGGGACGGTTCACATG
CAT	NM_009804	CCTGAGAGAGTGGTACATGC	CACTGCAAACCCACGAGGG
p53	NM_030989	GGCTCCTCCCCAACATCTTATC	TACCACCACGCTGTGCCGAAAA
HO-1	NM_012580	CGTGCAGAGAATTCTGAGTTC	AGACGCTTTACGTAGTGCTG
IL-1b	NM_031512	AGCTTCAGGAAGGCAGTGTC	TCCCACGAGTCACAGAGGA
TNF-a	AF269159	TCTGTGCCTCAGCCTCTTCT	GGCCATGGAACTGATGAGA
NF-kB	AF187319	AGACACAGAAGCACTACCTGACTC	GGCCCCACAATGTGTTGTA
VEGF-A	NM_031836	AAAAACGAAAGCGCAAGAAA	TTTCTCCGCTCTGAACAAGG
GAPDH	NM_008084	GACCCCTTCATTGACCTCAAC	CGCTCCTGGAAGATGGTGATGGG

### Protein expression analysis

After gel electrophoresis, proteins from the control, AI and VC groups were transferred onto nitrocellulose membranes. Membranes were blocked in 4% skimmed milk in TBST for 1 h, followed by serial incubation with HO-1 (Abcam, Cambridge, United Kingdom; 1:2000) and p53 (Abcam; 1:5000) antibodies at 4 °C overnight, a biotinylated goat anti-mouse whole IgG antibody (Amersham Biosciences, Freiburg, Germany; 1:10000) and finally, a streptavidin–HRP conjugate for 30 min (Amersham Biosciences). Bound antibodies were visualised using the ECL Plus Detection Reagent (Amersham Biosciences).

### Data analysis

Data were expressed as the mean ± SD. For statistical analyses, analyses of variance (two-way) and the Tukey–Kramer test were used. Statistical significance was set at *P* < 0.05 (two tailed).

## Electronic supplementary material

Supplementary Information

## References

[1] Harashina, T., Sawada, Y. & Watanabe, S. The relationship between venous occlusion time in island flaps and flap survivals. *Plast. Reconstr. Surg*. **60**, 92–95. Retrieved from http://www.ncbi.nlm.nih.gov/pubmed/20607944 (1977). 10.1097/00006534-197707000-00013 20607944

[2] Kobayashi S (2013). Changes of blood flow, oxygen tension, action potential and vascular permeability induced by arterial ischemia or venous congestion on the spinal cord in canine model. J. Orthop. Res..

[3] Su, C. T., Im, M. J. & Hoopes, J. E. Tissue glucose and lactate following vascular occlusion in island skin flaps. *Plast. Reconstr. Surg*. **70**, 202–205. Retrieved from http://www.ncbi.nlm.nih.gov/pubmed/7048369 (1982). 10.1097/00006534-198208000-00014 7048369

[4] Tsuchida, Y., Aoki, N., Fukuda, O., Nakano, M. & Igarashi, H. Changes in hemodynamics in jejunal flaps of rabbits due to ischemia, venous congestion, and reperfusion as measured by means of colored microspheres. *Plast. Reconstr. Surg*. **101**, 147–154. Retrieved from http://www.ncbi.nlm.nih.gov/pubmed/9427928 (1998). 10.1097/00006534-199801000-00024 9427928

[5] Yano, K., Hata, Y., Matsuka, K., Ito, O. & Matsuda, H. Time limits for intestinal ischemia and congestion: an experimental study in rats. *Ann. Plast. Surg*. **32**, 310–314. Retrieved from http://www.ncbi.nlm.nih.gov/pubmed/8192394 (1994). 10.1097/00000637-199403000-00015 8192394

[6] Damber JE, Bergh A, Daehlin L (1985). Testicular blood flow, vascular permeability, and testosterone production after stimulation of unilaterally cryptorchid adult rats with human chorionic gonadotropin. Endocrinology..

[7] Polguj, M., Jędrzejewski, K. S. & Topol, M. Arterial supply of human and bovine testes: a topographic and morphometric comparison study. *Folia Morphol*. **69**, 225–231. Retrieved from http://www.ncbi.nlm.nih.gov/pubmed/21120809 (2010). 21120809

[8] Visser, A. J. & Heyns, C. F. Testicular function after torsion of the spermatic cord. *BJU International*. **92**, 200–203. Retrieved from http://www.ncbi.nlm.nih.gov/pubmed/12887467 (2003). 10.1046/j.1464-410x.2003.04307.x 12887467

[9] Angel, M. F., Mellow, C. G., Knight, K. R. & O’Brien, B. M. The effect of deferoxamine on tolerance to secondary ischaemia caused by venous obstruction. *Br. J. Plast. Surg*. **42**, 422–424. Retrieved from http://www.ncbi.nlm.nih.gov/pubmed/2765735 (1989). 10.1016/0007-1226(89)90007-6 2765735

[10] Lysiak JJ, Zheng S, Woodson R, Turner TT (2007). Caspase-9-dependent pathway to murine germ cell apoptosis: mediation by oxidative stress, BAX, and caspase 2. Cell Tissue Res.

[11] Koji T (2001). Male germ cell death in mouse testes: possible involvement of Fas and Fas ligand. Med. Electron Micros.

[12] Lysiak, J. J., Turner, S. D. & Turner, T. T. Molecular pathway of germ cell apoptosis following ischemia/reperfusion of the rat testis. *Biol. Reprod*. **63**, 1465–1472. Retrieved from http://www.ncbi.nlm.nih.gov/pubmed/11058553 (2000). 10.1095/biolreprod63.5.1465 11058553

[13] Sukhotnik I (2009). Involvement of the bax and bcl-2 system in the induction of germ cell apoptosis is correlated with the time of reperfusion after testicular ischemia in a rat model. Fertil. Steril..

[14] Huang EJ, Kelly RE, Masuda H, Bjerke HS, Fonkalsrud EW (1990). Deleterious effects of testicular venous occlusion in young rats. Surg. Gynecol. Obstet..

[15] Bilgin-Karabulut A, Ademoğlu E, Aydin I, Erer M, Gökkuşu C (2001). Protective effects of vitamins A and E pretreatment in venous ischemia/reperfusion injury. J. Reconstr. Microsurg..

[16] Shridharani A, Owen RC, Elkelany OO, Kim ED (2016). The significance of clinical practice guidelines on adult varicocele detection and management. Asian J. Androl..

[17] Artyukhin, A. A. *et al*. Anatomy and microanatomy of the venous system of scrotal organs and spermatic cord. *Bull. Exp. Biol. Med*. **143**, 99–104. Retrieved from http://www.ncbi.nlm.nih.gov/pubmed/18019024 (2007). 10.1007/s10517-007-0027-9 18019024

[18] Baazeem A (2011). Varicocele and male factor infertility treatment: a new meta-analysis and review of the role of varicocele repair. Eur. Urol..

[19] Malekzadeh S (2016). Varicocele percutaneous embolization outcomes in a pediatric group: 7-year retrospective study. Int. Urol. Nephrol..

[20] Morimoto H (2013). ROS are required for mouse spermatogonial stem cell self-renewal. Cell Stem Cell.

[21] Morimoto H, Kanatsu-Shinohara M, Shinohara T (2015). ROS-Generating Oxidase Nox3 Regulates the Self-Renewal of Mouse Spermatogonial Stem Cells. Biol. Reprod..

[22] Scarabelli L (2011). Expression of antioxidant defense and poly(ADP-ribose) polymerase-1 in rat developing Sertoli cells. Cell Biol. Int..

[23] Takahashi T (2007). Heme oxygenase-1: a fundamental guardian against oxidative tissue injuries in acute inflammation. Mini Rev Med Chem.

[24] Budanov AV (2014). The role of tumor suppressor p53 in the antioxidant defense and metabolism. Subcell Biochem.

[25] Erol B (2017). Comparison of combined antioxidants and thymoquinone in the prevention of testis ischemia–reperfusion injury. Andrology.

[26] Sekmenli T (2016). The effects of melatonin and colchicine on ischemia–reperfusion injury in experimental rat testicular torsion model. J. Pediatr. Surg..

[27] Tunçkıran A (2005). Protective effect of vascular endothelial growth factor on histologic changes in testicular ischemia-reperfusion injury. Fertil. Steril..

[28] Mäkelä, E., Lahdes-Vasama, T., Rajakorpi, H. & Wikström, S. A 19-year review of paediatric patients with acute scrotum. *Scand. J. Surg*. **96**, 62–66, Retrieved from http://www.ncbi.nlm.nih.gov/pubmed/17461315 (2007). 10.1177/145749690709600112 17461315

[29] Mastrogiacomo, I. Immunological and clinical study of patients after spermatic cord torsion. *Andrologia*. **14**, 25–30, Retrieved from http://www.ncbi.nlm.nih.gov/pubmed/7039415 (1982). 10.1111/j.1439-0272.1982.tb03091.x 7039415

[30] Kalfa, N. *et al*. Ultrasonography of the spermatic cord in children with testicular torsion: impact on the surgical strategy. *J. Urol*. **172**, 1692–1695, discussion 1695. Retrieved from http://www.ncbi.nlm.nih.gov/pubmed/15371792 (2004). 10.1097/01.ju.0000138477.85710.69 15371792

[31] Kaye JD (2008). Parenchymal echo texture predicts testicular salvage after torsion: potential impact on the need for emergent exploration. J. Urol..

[32] Kühn AL, Scortegagna E, Nowitzki KM, Kim YH (2016). Ultrasonography of the scrotum in adults. Ultrasonography.

[33] Johnsen SG (1970). Testicular biopsy score count-a method for registration of spermatogenesis in human testes: normal values and results in 335 hypogonadal males. Hormones..

[34] Kanter M (2010). Protective effects of melatonin on testicular torsion/detorsion-induced ischemia-reperfusion injury in rats. Exp. Mol. Pathol..

[35] Kotani K, Yamada T (2012). Oxidative stress and metabolic syndrome in a Japanese female population. Australas. J. Ageing.

[36] Finotello R (2014). Redox status evaluation in dogs affected by mast cell tumour. Vet. Comp. Oncol..

[37] Menichini F (2015). Citrus medica L. cv Diamante (Rutaceae) peel extract improves glycaemic status of Zucker diabetic fatty (ZDF) rats and protects against oxidative stress. J. Enzyme Inhib. Med. Chem.

[38] Dohi K (2013). Status of systemic oxidative stress during therapeutic hypothermia in patients with post-cardiac arrest syndrome. Oxid. Med. Cell Longev.

