# Supplementary material for: Pathological effect of arterial ischaemia and venous congestion on rat testes
Source: Sci Rep. 2017 Jul 14;7:5422. doi: 10.1038/s41598-017-05880-2 (PMC5511218; doi:10.1038/s41598-017-05880-2)
Supplement: Supplementary file 1 — Supplementary Information [file 41598_2017_5880_MOESM1_ESM.pdf]

Supplementary Information

Pathological effect of arterial ischaemia and venous congestion on rat testes

Shuichi Hirai[1], Naoyuki Hatayama[1,2], Munekazu Naito[1], Kenta Nagahori[2], Shinichi Kawata[2], Shogo Hayashi[2], Ning Qu[2], Hayato Terayama[2,3], Sunao Shoji[4] and Masahiro Itoh[2]

1. Department of Anatomy, Aichi Medical University, 1-1 Yazakokarimata, Nagakute, Aichi 480-1195, Japan
2. Department of Anatomy, Tokyo Medical University, 6-1-1, Shinjuku, Shinjuku-ku, Tokyo 160-8402, Japan
3. Department of Anatomy, Division of Basic Medical Science, Tokai University School of Medicine, 143 Shimokasuya, Isehara, Kanagawa, Japan
4. Department of Urology, Tokai University Hachioji Hospital, 1838 Ishikawamachi, Hachiōji, Tokyo 192-0032, Japan

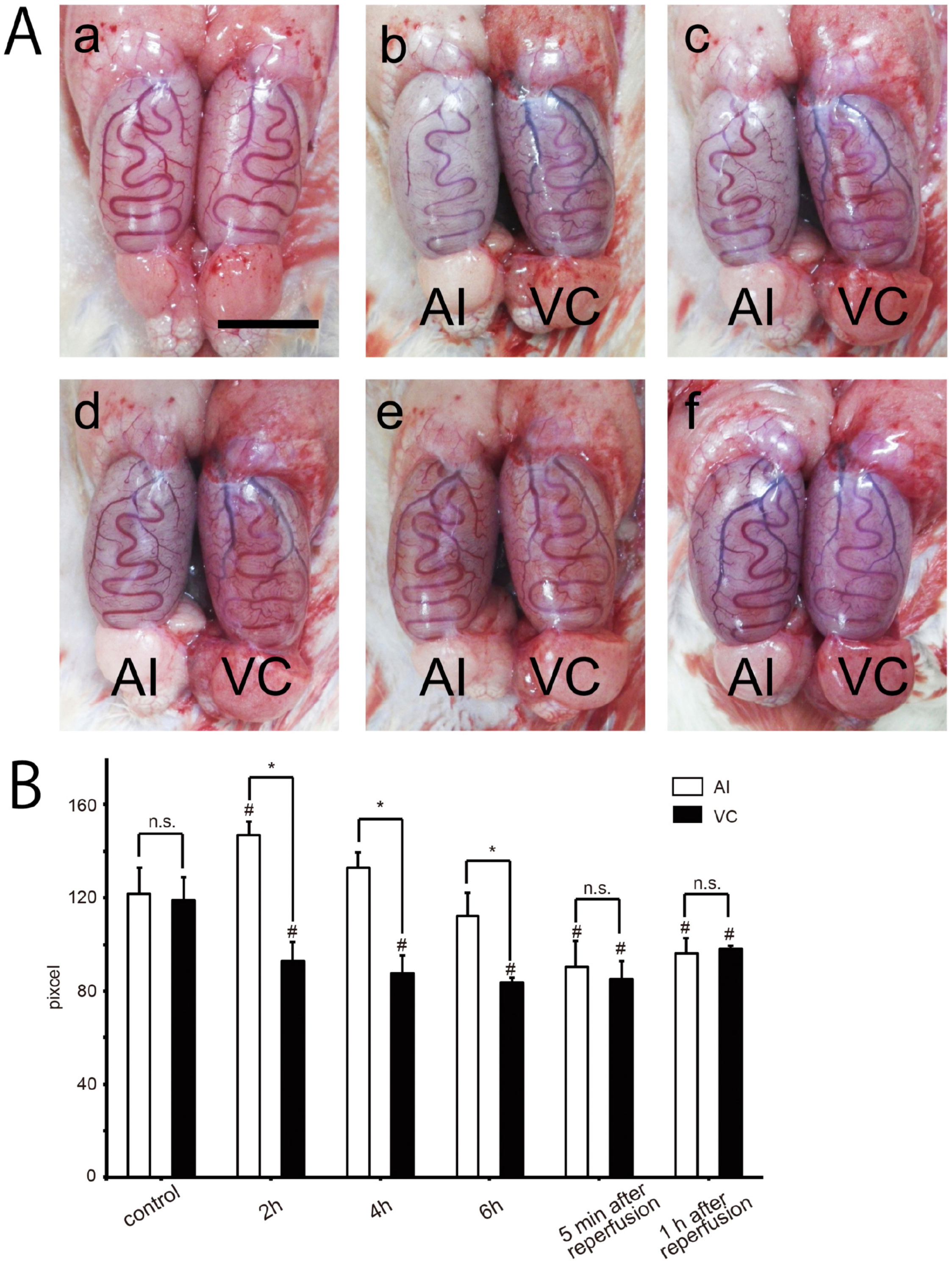

Supplemental Figure 1. Changes in the tissue color in rat testes with arterial ischemia (AI) and venous congestion (VC). A: Macroscopic findings. **a** Before blood flow interruption (BFI). **b** 2 h after BFI. **c** 4 h after BFI. **d** 6 h after BFI. **e** 5 min after reperfusion. **f** 1 h after reperfusion. Bar = 1 cm. B: Numerical conversion of brightness using histogram analysis. For color histogram analysis, the gray-scale histogram was constructed by converting each RGB image into a gray-scale value using the formula $V = 0.299R + 0.587G + 0.114B$ in Image J software (Image J version 1.48; NIH). The gray scale was constructed using the intensity per pixel area (as brightness values). Data are expressed as means ± SD, and the two-way analyses of variance and Tukey–Kramer tests were used for statistical analyses. $P > 0.05$ was considered as indicating nonsignificant differences. ns, not significant, # indicates $P < 0.05$ compared with the control. * indicates $P < 0.05$ for comparisons between AI and VC.

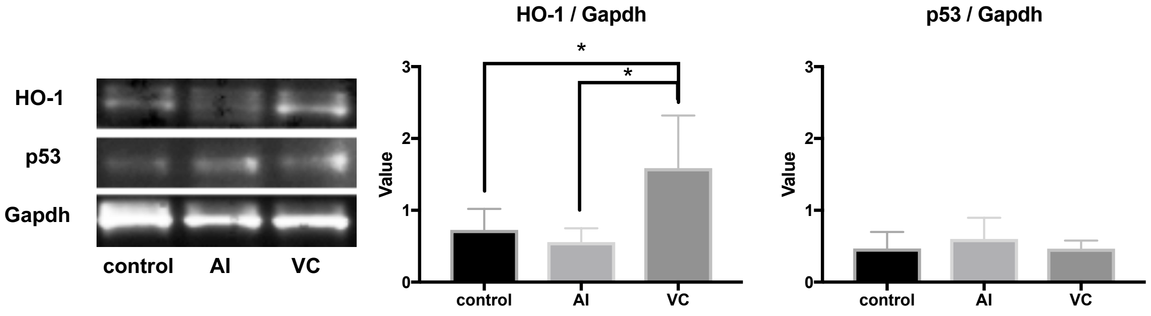

Supplemental figure 2. Protein levels of heme oxygenase (HO)-1 and p53 from rat testes with control, arterial ischemia (AI), and venous congestion (VC) at 6 h. Each bar represents the mean ± SD (n = 5). *$P < 0.05$

Supplemental table 1. Number of TUNEL-positive and PCNA-positive cells in ischemia (AI) and congestion (VC) groups.

Parameters		Control	2h	4h	6h
Number of TUNEL-positive cells / STs containing TUNEL-positive cells	AI group	0.1±0.5	2.0±0.5[a]	8.9±12.9[b]	15.4±15.9[c]
	VC group	0.1±0.5	0.1±0.4[d]	2.1±0.5[e]	2.5±7.1[f]
Number of PCNA-positive cells / STs containing PCNA-positive cells	AI group	51.8±10.2	50.2±12.9	25.61±24.6[g]	6.2±15.5[h]
	VC group	50.2±9.6	51.3±10.5	49.66±11.1[i]	35.96±21.3[j]

Values are expressed as the mean SD. p<0.05, a vs d, b vs e, c vs f, g vs i, h vs j
